# Doxorubicin leads to impaired insulin signaling in skeletal muscle

**DOI:** 10.1186/2049-3002-2-S1-P2

**Published:** 2014-05-28

**Authors:** Edson Alves de Lima, Camila Oliveira de Souza, Alexandre Abílio de Souza Teixeira, Helena Angélica Batatinha, Fábio de Santos Lira, José César Rosa Neto

**Affiliations:** 1 Department of Cell and Developmental Biology, ICB, São Paulo, São Paulo, Brazil; 2 Departament of Kinesiology, UNESP, Presidente Prudente, São Paulo, Brazil

## Background

Doxorubicin is an antibiotic largely used in clinical practice for the treatment of several types of tumors. However, its application may be limited by triggering some host’s deleterious effects, such as extreme fatigue, anorexia, sarcopenia, cardiovascular diseases, among others [1]. The role of skeletal muscle in this process should be further elucidated as this tissue is still important for glucose homeostasis [2] and becomes impaired by doxorubicin treatment. Knowledge of doxorubicin’s effect in the skeletal muscle and in the mechanism of action involved on the systemic insulin sensitivity is still incipient. The aim of this study is to evaluate the mechanism of insulin resistance after doxorubicin treatment.

## Materials and methods

Wistar rats received a single dose of intraperitoneal injection of doxorubicin (DOX) or saline (CT) at a dose of 15 mg/kg body. After 48h, some of the animals were submitted to evaluation of systemic insulin sensitivity by insulin tolerance test (iTT). After 72h the following parameters were analyzed: basal glycemia and insulinemia, mRNA expression of insulin pathway in EDL muscle (AKT, GLUT4, IRS-1, GSK3-b and AMPk) and activity of mitochondrial complexes, in EDL muscle. The groups were compared by Test T. p < 0.05 was considered statistically significant.

## Results

After 72 hours, the Dox group showed a decrease in body weight and food intake. Insulin sensitivity assessed by ITT was decreased in the DOX group, corroborating the data of systemic resistance assessed by elevated basal insulin and glucose. There was an increase in the mitochondrial complex 1 activity and a decrease in complex 3 activity. mRNA expression of the insulin signaling pathway in skeletal muscle was altered by treatment. The DOX group showed a decrease of IRS-1, GLUT4, AMPK and GSK3b expression. Gene expression of AKT was not altered.

## Conclusions

Our data suggests that doxorubicin causes systemic insulin resistance and also alters the expression of genes involved in insulin signaling in skeletal muscle. AMPK may be responsible for one of the paths of insulin resistance development in the DOX group.
